# Somatic genomics as a discovery engine for biomedicine

**DOI:** 10.1016/j.cell.2026.01.032

**Published:** 2026-03-05

**Authors:** Simon F. Brunner, Iñigo Martincorena, Gregory Mannino, Caroline S. Fox, Michael R. Stratton, Jacob R. Rubens, Peter J. Campbell, Hao Zhu

**Affiliations:** 1 Quotient Therapeutics, Inc., Cambridge, MA, USA; 2 Flagship Pioneering, Inc., Cambridge, MA, USA; 3 Wellcome Sanger Institute, Wellcome Genome Campus, Hinxton, UK; 4 Children’s Research Institute, Departments of Pediatrics and Internal Medicine, Simmons Comprehensive Cancer Center, Center for Regenerative Science and Medicine, University of Texas Southwestern Medical Center, Dallas, TX, USA; 5 Work done while at Quotient Therapeutics

## Abstract

Somatic mutations, or genetic changes occurring in cells after conception, are widespread in healthy tissues but are conventionally viewed as signs of pre-cancer or simply a consequence of aging. However, an emerging body of work has shown that somatic mutations can drive or protect against disease, which could inspire novel therapeutic strategies. The unexpected depth of genetic diversity within individuals also provides a massive substrate for discovering mutant genes selected for by disease. For instance, mutant hematopoietic cells can exacerbate inflammatory disease, and mutant hepatocytes can protect against liver disease. This suggests that somatic mutations, whether maladaptive or beneficial, could provide crucial insights into disease mechanisms, history, and reversal strategies. Somatic genetics offers a powerful, complementary approach to traditional germline genetics, which has had an enormous impact on biomedicine and drug development. This review explores the factors that shape the landscape of somatic mosaicism and discusses somatic mutations that cause or protect from disease. We highlight how somatic mutations are becoming a key discovery engine for disease genetics, moving rapidly toward drug target identification and clinical translation.

## INTRODUCTION: SOMATIC MUTATIONS ARE WIDESPREAD IN HEALTH AND DISEASE

Somatic mutations can occur at any stage of development, and those that occur early in embryogenesis can lead to striking examples that illustrate the potency of these mutations. In Proteus syndrome, a large fraction of cells in affected individuals contain activating mutations in *AKT1* or inactivating mutations in *PTEN*, leading to debilitating limb and organ overgrowths. ^1,2^ Arteriovenous malformations (AVMs), or large vascular tangles that can arise in any part of the body, are frequently caused by somatic mutations in phosphatidylinositol 3-kinase (*PI3K*) or *KRAS*. ^3,4^ Café au lait macules, associated with neurofibromatosis, are mosaic hyperpigmented skin lesions that are caused by loss of *NF1* heterozygosity. Autosomal dominant polycystic kidney disease is a germline syndrome whereby innumerable cysts form after the somatic loss of a second copy of *PKD1* or *PKD2*. ^5^ While these syndromes are morphologically striking, perhaps the most devastating examples of somatic mosaicism occur in sporadic cancers. Somatic mutations are necessary for the development of most cancers, and a major effort in modern oncology seeks to design treatments based on the genes that are somatically mutated in cancers.

In healthy individuals, mutations accumulate steadily in all somatic cells throughout life. ^6,7^ Most of this spontaneous genetic variation is driven by endogenous mutational processes that arise from the standard biochemical processes of cellular function, such as spontaneous deamination of methylated cytosines, DNA replication or repair errors, and reactive oxygen species. These endogenous mutational processes begin from the moment of fertilization and continue throughout life, even in post-mitotic cells (Figure 1). ^8^ As a result, the rate of mutation accumulation is generally constant over time such that there is a linear correlation between age and mutation burden in most tissues. On average, each adult has approximately 1,000–2,000 mutations per cell and accumulates 20–50 new mutations/cell/year (Figure 1). However, there is up to 10-fold variation in mutation burden across different somatic organs, resulting from tissue-to-tissue and cell-to-cell variation in the activity of endogenous mutational processes. ^9–11^ Tissues subject to additional exogenous mutagen exposures, such as skin (sun), ^12^ liver (alcohol), ^13,14^ lung (pollution, tobacco), ^15^ esophagus (alcohol and tobacco), ^16^ and bladder (alcohol and tobacco), ^17^ harbor increased mutation burden.

While most mutations do not change the cell’s phenotype and are functionally inconsequential, some will inevitably land in critical coding regions or regulatory elements of the genome and exert phenotypic effects. Such phenotypic variation is the substrate upon which Darwinian selection can act, resulting in the disappearance or expansion of clones with altered fitness. As a consequence, normal tissues from healthy individuals can show the emergence of clones that carry positively selected somatic mutations, so-called “driver” mutations. By the age of 70 years, about a third of sun-exposed skin cells, ^12^ about 50% of esophageal epithelial cells, ^16^ and about 60% of endometrial gland cells ^19^ will carry at least one driver mutation. In many organs, these driver mutations overlap between normal and malignant tissues, suggesting that the earliest events in cancer evolution are already detectable in normal tissues. However, many driver mutations in normal tissues are not seen, or are seen with reduced frequency in cancers, ^16,20^ and therefore do not participate in transformation. Disease processes represent additional selective pressures on mutant clones. The landscape of somatic mutations has been studied for conditions such as chronic liver disease, ^13,14,21^ inflammatory bowel disease (IBD), ^20,22,23^ psoriasis, ^24^ and a small number of monogenic germline disorders. ^25–27^ Several general concepts emerge from these early studies: (1) diseases often select for a different repertoire of driver mutations than seen in healthy tissues and cancers, (2) clones often grow to larger sizes in disease tissue than seen in healthy tissues, and (3) convergent selection of the same driver mutations within and across patients is frequent. We will devote much of this review toward a discussion of somatic mutations identified in disease and what they might mean for clinical translation.

## INTRINSIC AND EXTRINSIC FACTORS THAT SHAPE THE LANDSCAPE OF SOMATIC MOSAICISM

### Tissue architecture and proliferation regulate somatic mosaicism

Just as geographic factors play a crucial role in speciation, ^28^ the process by which new biological species arise, organ-specific architecture could also determine the degree of somatic diversity. This topic is in the early stages of exploration ^29^ but could lead to predictions about the size of mutant clones, the number of genes under selection, and the overall genetic diversity within an organ. The hematopoietic system has few geographic and space limitations, and blood mutations that confer the greatest fitness benefits can expand without spatial limits. Similarly, the skin and esophagus contain sheets of squamous tissues in which somatic mutant clones can expand two-dimensionally without restricted borders. These less restrictive boundaries allow for larger clone sizes and variant allele frequencies (VAFs). ^29,30^ Liver lobules provide an interesting contrast since hepatocytes are constrained by lobular boundaries, which become fibrotic barriers in chronic disease. Similarly, the intestinal crypt-villus structure limits the degree of stem cell expansion across the tissue. ^31^ These gastrointestinal tissues contain innumerable clones that would be predicted to allow for increased genetic diversity and divergent evolutionary outcomes. ^30^ In contrast with clonal hematopoiesis, where the same genes are recurrently mutated in most patients, ^32,33^ cirrhotic livers show high genetic diversity, ^13,14,21^ where sampling of different sites within a single liver can reveal disparate genes under selection. Similarly, squamous tissues like skin and esophagus, with fewer geographic constraints, demonstrate dominance of *NOTCH1* and *TP53* mutant clones. ^12,16^ Other tissue-intrinsic variables certainly contribute to somatic genetic diversity. The degree of cell turnover within a tissue can influence diversity, with blood and intestine possessing rapid turnover, while the brain is predominantly post-mitotic. As discussed, increased mutagenesis rates in tissues such as the liver could also contribute to clonal diversity. Strong extrinsic pressures that lead to positive or purifying selection can lead to reduced genetic diversity and are discussed below. Mutagenesis, clonal selection, and architectural constraints can operate together to shape somatic evolution. ^34^ To isolate how architecture contributes to the somatic mutation landscape, it will be important to theoretically and experimentally model somatic evolution in tissues with unrestricted vs. restricted structures.

### Inflammation shapes and is shaped by somatic mosaicism

Selective pressures are evolutionary forces that influence the survival of clones, and new selective pressures may arise during aging or disease pathogenesis. Inflammation is one of the selective pressures that increase clonal hematopoiesis of indeterminate potential (CHIP). CHIP, defined as the presence of a clonal population comprising more than 4% of the peripheral blood, is present in more than 15% of individuals in their 70s and is associated with increased risks of leukemia, ^32,33^ cardiovascular disease, and ischemic stroke. ^35^ While CHIP is strongly associated with these and other inflammatory conditions, it has been very difficult to establish causality based on sequencing data alone. Some insights into causality have been gained through mouse models. While CHIP may drive atherosclerosis, there is also mathematical and experimental evidence indicating that atherosclerosis might cause clonal expansions in CHIP. ^36^ In rodent models, inflammation has been shown to promote clonal expansion of *TET2* mutant cells through interleukin (IL)-6 and tumor necrosis factor α (TNF-α) activity. ^37,38^ Mycobacteria, which produce interferon gamma (INFg), can cause expansion of *DNMT3A*-deficient multipotent progenitors in murine models, thereby demonstrating that inflammation from an infectious source can promote the emergence of CHIP. ^39^ In turn, Wang et al. showed that in mouse models, *DNMT3A* mutations in CHIP caused increased periodontal disease and arthritis through increased osteoclastogenesis and IL-17. ^40^ In tandem with human CHIP studies, experimental models suggest that inflammation and CHIP are ensnared in a complex feedback loop, with inflammation driving CHIP and CHIP promoting inflammation.

Inflammation may also impact the landscape of somatic mutations in solid organs. Intestinal epithelial cells in ulcerative colitis patients have more somatic mutations compared with healthy controls. ^20,22,23^ Even within an individual ulcerative colitis patient, cells in the descending colon, which is more inflammatory, harbor more somatic mutations than cells in the ascending colon. ^20,22^ Similarly, chronic inflammation is also associated with increased somatic mutations in gastric epithelial cells. ^41^ Conversely, in Barrett’s esophagus patients, fewer somatic mutations were found in those who continually used non-steroidal anti-inflammatory drugs. ^42^ Despite experimental evidence, the degree to which somatic mosaicism (i.e., CHIP) is the cause of inflammatory diseases and vice versa is uncertain, illustrating the challenge of understanding somatic mutations and their relationships with disease.

### Chemicals, toxins, and chemotherapies are selective pressures that mediate clonal expansion

Some chemicals act as carcinogens by increasing mutagenesis, but carcinogens can also select clones through “promoter” mechanisms. Many carcinogens, originally thought of as mutagens, likely promote cancer through altering the selective landscape experienced by tissues, thereby facilitating clonal expansion. ^43^ In lung cancer, pollution could be promoting cancer initiation by increasing inflammation through IL-1β, which leads to the expansion of pre-existing mutant *KRAS* clones. ^44^ The larger the clone, the greater the likelihood of a second hit mutation that drives malignant transformation. Similarly, smoking and alcohol are associated with increased mosaicism in the non-malignant lung, ^15^ liver, ^14^ esophagus, ^16^ and bladder, ^17^ likely in part by altering selective pressures (Figure 2). Another example is therapy-related clonal hematopoiesis, which is CHIP that is observed in patients treated with chemotherapy or radiation. In addition to creating new mutant clones, chemotherapy likely causes the expansion of pre-existing mutant clones. ^45–48^ Altogether, many exogenous agents shape the somatic mutation landscape not through mutagenesis, but instead through increasing cell turnover, creating niche space for regenerative clones, or by exerting new selective pressures. From an adaptive viewpoint, mutations might point to mechanisms of resistance or enhanced regeneration after exposure to promoter agents. An extension of this concept is that long-term exposures to medications could lead to altered selection of somatic clones that may provide useful clinical information.

### Metabolic disease shapes somatic mosaicism in the liver and esophagus

In modern society, a widespread exposure that underlies an extraordinary burden of disease is overnutrition, and it stands to reason that this would also shape somatic mosaicism. Chronic liver disease is an exemplar of how different environmental pressures could select for distinct somatic mutations. The most common etiologies are viral hepatitis (hepatitis B and hepatitis C), chronic alcohol consumption, and metabolic dysfunction-associated steatotic liver disease (MASLD) (Figure 2A). These processes induce cycles of cell death and regeneration that over decades lead to liver cirrhosis, a clinical state where liver nodules are surrounded by bands of fibrosis. The combination of chronic toxicity and regeneration provides a trove of somatic mutations that influence clonal dynamics. Two groups reported somatically acquired driver mutations in regenerative nodules in chronic liver disease. ^13,14^ In a cohort of predominantly hepatitis C-associated cirrhosis patients, driver mutations in *PKD1*, *ARID1A*, and *KMT2D* were identified. In a cohort of alcohol-related or MASLD patients, driver mutations in *FOXO1*, *GPAM*, and *CIDEB* were found. Strikingly, the genes in MASLD are not classic cancer drivers, and the mutations are predicted to modulate the storage, synthesis, or oxidation of lipids in affected hepatocytes.

The MASLD studies demonstrated convergent evolution within patients but considerable divergence between patients, which could suggest that selective pressures differ between patients. A given patient with MASLD will typically show convergent evolution of multiple independent clones that carry mutations in the same driver gene. This convergence can lead to hundreds of grams of liver tissue affected by mutations in the same driver gene, indicating the potential to have organ-wide functional consequences. In support of this, murine studies show that loss of *Pkd1* in just 5% of the liver can lead to measurable metabolic benefits at the organismal level. ^49^ Intriguingly, patients often differ in which genes are mutated—one might have many *FOXO1*-mutant clones, another may harbor *GPAM-*mutant clones, and a third could show a predominance of *CIDEB* mutations. The disease process in a given patient introduces a distinctive, organ-wide selective pressure that a limited repertoire of mutations can alleviate—this causes the within-patient convergence of drivers. In another patient, the disease process might introduce a different selective pressure, for which a different set of driver mutations emerges—this causes the between-patient divergence in mutations (Figure 2A). For example, one patient might arrive at MASLD through excess alcohol and carbohydrates, in which case hepatocytes with mutations targeting *de novo* lipogenesis genes (such as *GPAM* ^50^ ) would be more likely to survive and regenerate. Another patient might arrive at MASLD through dietary excess of fats, in which case *de novo* lipogenesis would be less important, but genes involved in lipid storage and oxidation, such as *CIDEB*, ^51,52^ might be more beneficial. Thus, the set of driver genes illustrates the genes and pathways that provide protection when mutated. Furthermore, the patterns of driver mutations observed in different patients provide clues to the subtypes of disease processes in the population and may be useful as companion diagnostics for targeted therapeutics. Experimental evidence for this concept was demonstrated for *CIDEB*, where loss-of-function mutations were more positively selected under some but not all diets that promote fatty liver disease. ^51^

Other tissues also harbor undesirable mutations whose selection is sensitive to metabolic stresses. In the esophagus, there is a high frequency of *PIK3CA* mutations (Figure 2B). Herms et al. modeled *Pik3ca* ^*H1047R*^ clones using knockin heterozygous mice and demonstrated that squamous clones containing these mutations expand with age by outcompeting wild-type (WT) clones. ^53^ Inducing diabetes contributed to the growth of *Pik3ca* mutant clones because insulin-resistant WT cells are less able to respond to circulating insulin, whereas mutant cells are less dependent on circulating insulin for pathway activation. Conversely, metformin treatment reduced the size of these clones. This elegantly demonstrated that environmental conditions such as obesity and diabetes can lead to the positive selection of mutant vs. WT clones. This could be one mechanism by which obesity promotes pre-cancer growth in some tissues.

## SOMATIC MUTATIONS CAN DRIVE DISEASE

Somatic mutations are emerging as unexpected drivers of diseases with previously idiopathic etiologies, i.e., focal epilepsies and autoimmunity. Disease-driving genes with clear pathogenic mechanisms represent actionable therapeutic targets (Figure 3; Table 1). These salient examples should stimulate the search for other somatic mutations that cause non-malignant diseases.

### Somatic mutations are implicated in diverse neuropathologies

Mutations in the brain are some of the best examples of how a small number of mutated cells can have a profound impact on disease. Malformations of cortical development (MCDs) are a group of neurological disorders caused by improper development of the cerebral cortex. MCDs, driven in large part by somatic mutations acquired during development, often present with intractable epilepsy. Chung et al. found one or more somatic mutations in 76 of 283 (27%) MCD patients. ^54^ These most frequently involve activating mutations in the PI3K-AKT-mTOR pathway (Table 1), and the clone sizes correlate with the developmental stage of the mutation and the magnitude of the phenotype. For example, activating mutations in *AKT3* with a VAF of 10%–30% can cause hemimegalencephaly, or overgrowth of an entire hemisphere. ^55^ MCDs with smaller lesions (VAF of <5%) are clinically categorized as focal cortical dysplasia (FCD), a cause of drug-resistant focal epilepsy and the most common reason for epilepsy surgery in children. Activating mTOR mutations with VAFs of less than 1% can be observed in FCD patients. In murine models, mTOR mutations in as few as 8,000 neurons (constituting 0.04% VAF) were sufficient to induce epileptic seizures,^56^ demonstrating that somatic mutations with very low frequencies can induce neuropathology. These focal epilepsies are not restricted to children, as somatic mutations in *RAS*/*RAF*/mitogen-activated protein kinase (*MAPK*) can lead to mesial temporal lobe epilepsy in adults. ^57^ Recent studies have also connected somatic mutations in the brain to non-epileptic psychiatric disorders such as autism spectrum disorder.^58^ Maury et al. sequenced neurons from schizophrenia vs. neurotypical control patients and found an increased rate of single-nucleotide variants (SNVs) in transcriptionally relevant sites in disease, supporting the functional relevance of these variants. ^59^ A recent study of over 10,000 participants found an increase in somatic copy-number variants in schizophrenia patients vs. controls and also revealed recurring deletions in the *NRXN1* and *ABCB11* genes.^60^

### Somatic mutations can cause vascular, lymphatic, and limb malformations

AVMs are vascular anomalies where arteries and veins are inappropriately connected without the presence of a normal capillary bed. They can occur in any organ, but AVMs in the brain or spinal cord are most challenging due to the potential for uncontrolled bleeding. AVMs are often caused by somatic activating variants in the RAS-MAPK pathway (i.e., *MAP2K1*, *KRAS*, *BRAF*, and *RIT1*). ^61–64^ Targeted therapies against mutant MEK and ERK are being tested in AVM patients, in part based on preclinical signals. ^65,66^ Similarly, lymphovascular malformations (LVMs) are congenital lymphovascular anomalies characterized by dilated and fluid-filled cystic lymphatic channels. These can be disfiguring when found in the head and neck area but can also affect the skin and soft tissues in virtually any part of the body. These malformations are often caused by somatic mutations in *PIK3CA*, ^67,68^ which represents a compelling therapeutic target in both AVMs and LVMs. Ollier disease and Maffucci syndrome are non-hereditary skeletal disorders characterized by enchondromas and hemangiomas. Both are associated with somatic mutations in *IDH1* or *IDH2*, ^69–71^ and positive clinical responses to IDH1 inhibitors have been reported. ^72^

### Somatic mutations can drive autoimmune and autoinflammatory conditions

Somatic STAT3 gain-of-function mutations are causal of the autoimmune aspects of Felty syndrome and T cell large granular lymphocyte leukemia. ^73^ Murine studies show that these mutations are the driver of CD8 ^+^ effector-mediated autoimmunity and not simply a consequence of chronic disease. Somatic and germline *NLRP3* mutations drive an autoimmune disease called chronic infantile neurologic cutaneous articular (CINCA) syndrome, which is characterized by rash, fevers, meningitis, and arthritis. ^74^ Somatic *KRAS* mutations have been identified in small cohorts of patients with autoimmune disorders that resemble lupus, although the causal connections are uncertain. ^75^ Somatic myeloid-restricted mutations in *UBA1* have been found in patients with vacuoles, E1 enzyme, X-linked, autoinflammatory, somatic syndrome (VEXAS), a constellation of findings that include fever, rash, and inflammation, anemia, and low platelets. ^76^ These examples, many of which may nominate promising drug targets, are likely just the initial discoveries that link somatic mutations with a broad array of rheumatic disorders.

A more pervasive causal link between somatic mutations and autoimmune conditions beyond rare syndromes may exist. Phylogenetic reconstruction of self-reactive B cells in cryoglobulinemic vasculitis demonstrated that driver mutations in lymphoma genes such as *CARD11*, *TNFAIP3*, and *KLHL6* occur early in B cell clonal expansions,^77^ potentially leading to B cell clones that can evade “self” checkpoints or anergy. More recently, the same group proposed a mechanistic basis for the emergence of cryoglobulinemic vasculitis in hepatitis C virus (HCV)-infected patients.^78^ According to their model, pathogenic B cell clones arise via a three-hit process: (1) common germline V(D)J rearrangements generate a public rheumatoid factor precursor repertoire of low-affinity, self-IgG-binding B cell receptors; (2) off-target, genome-wide somatic mutations (SNVs, indels, structural variants [SVs], or chromosome gains/losses) hit lymphoma-driver loci (e.g., *KLF2*, *NOTCH1*, and *TRAF3*) to confer increased survival and expansion; and (3) activation-induced cytidine deaminase (AID)-mediated somatic hypermutation in the Ig variable regions introduces affinity-enhancing replacements that convert these clones into high-avidity, cryoprecipitating autoantibody-secreting cells. If true, this mechanism could plausibly be involved in many autoimmune diseases, elucidate triggers that give rise to checkpoint-evading mutant clones, and provide strategies to inhibit autoreactive immune cells.

## SOMATIC MUTATIONS CAN PROTECT AGAINST OR COUNTER DISEASE

One of the most exciting recent conceptual advances for somatic mosaicism is that there are many adaptive mutations that protect against or counteract chronic diseases (Figure 3). These can be seen as treatment opportunities, much like analogous examples from germline genetics such as *PCSK9* for hypercholesterolemia ^79,80^ or *NAV1.8* for pain.^81 ,82^ Some of the following examples suggest adaptive functionalities that are worth mimicking with therapeutic approaches. Environmental shifts or changes in selective pressures associated with disease can mediate the selection of somatic mutations that become advantageous in a contingent manner. Thus, the adaptive or beneficial nature of mutations must be interpreted in light of the appropriate disease context.

### *NOTCH* mutations in the esophagus compete against cancer

*TP53* and *NOTCH1/2* mutations can occupy a considerable fraction of squamous cells in the skin, esophagus, and oral epithelium. ^12,16,24^ While *TP53* mutations are common in both non-malignant esophagus and in squamous cell carcinomas derived from the esophagus, *NOTCH1/2* mutations are more prevalent in normal tissues than they are in cancer. This suggests that these mutations could be passengers and not drivers within cancer. Experimentally, *Notch1* mutations in normal squamous cells exert competitive effects against early esophageal tumors. ^83,84^ Taken together, it is plausible that *NOTCH1* mutations are preventing cancer initiation in the esophagus. While the loss of *NOTCH1* is not unconditionally adaptive, these statistical and experimental examples suggest that *NOTCH1* mutations could have pleiotropic effects in the aging esophagus that demand further investigation.

### Somatic mutations in liver disease protect against tissue injury

While germline genetics is a powerful way to identify human genes involved in disease development, it has not been used to identify human genes involved in tissue regeneration. This is in part because there is no blood test or quantitative trait for regeneration, and humans with exceptional regeneration would not come to medical attention. As a source of genetic heterogeneity in regeneration, somatic mosaicism could be used to identify new pathways in regeneration and wound healing. For example, adaptive somatic mutations have been identified in the cirrhotic liver. Somatic mutations in *PKD1*, *KMT2D*, and *ARID1A* can increase cellular fitness and protect cells from injuries, leading to the expansion of adaptive clones.^13^ The adaptive nature of these mutations was made clear by generating mouse models containing loss-of-function heterozygous mutations in the entire liver. Mutations in *PKD1*, *KMT2D*, or *ARID1A* could promote regeneration in the liver after insults such as surgical resection or chemical toxins.

Intriguingly, the adaptive nature of hepatic somatic mutations extends beyond proliferation and regeneration. The abnormal accumulation of lipid droplets in hepatocytes can eventually lead to lipotoxicity, cell death, inflammation, and fibrosis in steatotic liver disease. The observation that loss-of-function mutations in lipid metabolism genes such as *GPAM* and *CIDEB* and hotspot mutations in *FOXO1* are enriched in MASLD ^21^ suggests that some somatic mutations confer increased fitness through disrupting lipogenesis pathways. In mouse models of somatic mosaicism, loss-of-function *Gpam* or *Cideb* mutant cells are positively selected more than WT cells within fatty livers. ^51,85^ Somatic genetic studies in humans and mice together establish the concept that positive selection of mutant clones in mosaic tissues can identify new metabolic disease regulators. Multiple pharmaceutical companies are pursuing treatments that capitalize on these protective insights, leading to clinical investigation of CIDEB inhibitors (NCT06836609) and underscoring the therapeutic potential of somatic genetics.

### IL-17 resistance mutations may exacerbate colitis but inhibit transformation

In IBD, multiple groups simultaneously identified highly recurrent somatic mutations in IL-17 signaling genes within intestinal tissues. ^20,22,23^ Loss-of-function mutations were found in *NFKBIZ* and *PIGR*, and gain-of-function mutations were found in *ZC3H12A*. Experimental modeling of these mutations showed that they rendered intestinal cells resistant to the detrimental effects of IL-17-mediated inflammation. While these mutations can protect individual clones, ^22^ it is not yet clear how the net effect of clonal expansions impacts the progression of IBD in individual patients. IL-17 inhibition or *Pigr* deletion in mice, which mimics the directionality of the human mutations, results in worse colitis. ^86–88^ These results agree with the clinical observation that IL-17 inhibitors (secukinumab and brodalumab) worsened outcomes in IBD trials. ^89,90^ By contrast, Kakiuchi et al. showed that *Nfkbiz*-mutant clones expand *in vivo* but did not affect the severity of intestinal inflammation in mouse models. ^20^ Furthermore, *NFKBIZ* loss-of-function mutations are rare in colon cancer, and intestinal tumor formation was robustly suppressed in *Nfkbiz*-mutant mice. It is possible that IL-17-resistant mutant clones might exacerbate colitis but ultimately suppress inflammation-associated carcinogenesis.

### Mutations in the immune system can promote anti-cancer immunity

CHIP mutations appear to exacerbate various diseases and portend leukemogenesis but could be protective in some contexts. Given the strong connection between CHIP, inflammation, and immunity, it is unsurprising that somatic mutations in myeloid or lymphoid populations play functional roles in cancer-related immunity. Within non-small cell lung cancers, the presence of immune cells containing CHIP mutations is strongly associated with an increased risk of death and recurrence. ^91^ However, in the setting of bone marrow transplant, CHIP in the donor marrow can benefit the recipient in the form of reduced relapse rates and increased survival. ^92^ In this setting, *DNMT3A* mutant cells mount a stronger immune response against residual leukemia cells in the recipient. In contrast to the situation in non-immunotherapy-treated patients, ^91^ Hsiehchen et al. also showed that CHIP is associated with improved immunotherapy responses in a subset of cancer types. ^93^ In support of this clinical study, Rondeau et al. showed in mouse models that *TET2* mutations in the blood synergize with immune checkpoint blockade to inhibit solid cancer growth in a CD4/CD8/phagocyte-dependent manner. ^94^ The mechanistic basis of these context-specific benefits of somatic mutations requires further investigation.

Less commonly known mutations in the blood also play a role in immunity and immunotherapy response. Mutations identified in lymphomas have recently been shown to confer strong anti-cancer effects when introduced into CAR-T cells. ^95^ Garcia et al. screened 71 mutations from T cell neoplasms and identified a gene fusion, *CARD11-PIK3R3*, that augments anti-tumor efficacy of therapeutic T cells in immunotherapy-refractory models. In another study, whole-genome sequencing of tumor-infiltrating lymphocytes (TILs) from four patients with melanoma identified *SH2D2A* and *TNFAIP3* loss-of-function mutations. ^96^ *TNFAIP3* loss activated T cell effector functions *in vitro* and in mice. A recent study using whole-exome duplex nanorate sequencing (NanoSeq) of TILs identified activating mutations in *TYK2*, a JAK family member.^97^ To assess phenotypic impact, a germline *Tyk2* ^*D807V*^ mutation that recapitulates the human *Tyk2* ^*D810V*^ mutation was knocked into mice. While *Tyk2* ^*D807V*^ mutant mice did not develop autoimmune pathologies, they possessed enhanced anti-tumor effects in the context of multiple syngeneic and autochthonous cancer models. Clinical translation of the above findings could involve genetic modifications of CAR-T cells or small molecules that mimic these mutations. These modifications could in theory increase the risk of cancer development, and thus careful selection of somatic mutations that are less likely to drive cancer will be needed. ^98,99^

### Somatic variants can counteract the detrimental effects of monogenic diseases

Germline mutations that cause monogenic diseases also exert selective pressures from which adaptive somatic mutations can arise. In these settings, somatic mutations can counteract germline mutations to promote clonal fitness. One such example is observed in telomerase disorders in which patients develop pulmonary fibrosis, skin abnormalities, bone marrow failure, or premature aging. In these patients, somatic reversion mutations in the *Telomerase* promoter appear to counteract disease phenotypes caused by loss-of-function coding mutations in telomere pathway genes. ^100^ Since the germline mutations are heterozygous, these promoter mutations are thought to increase *Telomerase* expression from the WT allele. Ultimately, this is believed to increase clonal fitness of hematopoietic stem cells that lack normal telomerase function. In Shwachman-Diamond syndrome (SDS), germline mutations in *SBDS* or *EFL1* lead to a defect in ribosome biogenesis. Normally, these proteins release EIF6 from the 60S ribosomal subunit, an essential step for ribosomes to translate proteins. In SDS, persistent binding of EIF6 leads to reduced ribosomal maturation and decreased protein synthesis. Several groups identified somatic *eIF6* “rescue” mutations that lead to reduced EIF6 levels or lowered affinity for the 60S subunit. ^25,26,101^ These mutations counter the negative effects of the original SDS mutations and effectively nominate EIF6 as a therapeutic target in SDS.

Other examples have been detected in the liver, where mutant hepatic clones enlarge due to increased regeneration within a germline disease. Hereditary tyrosinemia is an inborn error of tyrosine metabolism caused by fumarylacetoacetate hydrolase (*Fah*) deficiency and leads to chronic liver disease. In *Fah* knockout (KO) mice, chromosomal loss or missense mutations that inactivate homogentisic acid dioxygenase (Hgd) ameliorate the toxic effects of tyrosine precursor buildup and manifest as healthy nodules that emerge within a cirrhotic liver. ^102,103^ In humans with alpha-1 antitrypsin (A1AT) deficiency, the disease-causing Z (E342K) allele, located at the C-terminal domain of A1AT, leads to protein aggregation. ^104^ Brzozowska et al. identified somatic variants that ameliorate the liver pathology caused by this germline defect. Acquired truncation mutations in *SERPINA1*, the gene encoding A1AT, are clustered in the vicinity of the Z-allele and are positively selected for in A1AT-deficient human livers. In functional studies, C-terminal truncation *SERPINA1* variants reduce disease-associated Z-A1AT polymer accumulation, thus ameliorating the effects of disease within mutant clones. ^27^ These disease-specific variants suggest that genetic solutions can be identified within the tissues of monogenic disease patients. Since gene therapy-based correction of germline mutations is currently restricted in terms of tissue types and patient access, more easily drugged alternative targets such as the ones discussed above could address unmet needs.

### Somatic mutations can have divergent influences on clones and individuals

A major challenge in the field is understanding the effects of mutant clones on the organismal level. Unlike germline variants, it is difficult to predict or measure the phenotypic impact of low VAF mutant clones on individual patients. Complex influences of clones on organisms that are not purely detrimental or adaptive may represent the rule and not the exception. Discord between clone fitness and organismal fitness is important but difficult to identify. Protective mutations in the liver provide clear examples of how increased clonal fitness would translate into increased organismal fitness, especially when the genes are well understood or have known germline genotype-phenotype relationships. However, variants in less-understood genes can cause increased clonal fitness that might even lead to increased organ fitness, but at the cost of reduced organismal fitness. For example, hepatocyte clones harboring somatic mutations in the transcription factor *TBX3* are positively selected in mice on MASLD diets because they reduce lipid accumulation and lipotoxicity through increased VLDL secretion. ^105^ Consistently, liver-wide *Tbx3* deletion protects against diet-induced MASLD. However, the increased VLDL secretion causes exacerbated hyperlipidemia and insulin resistance, ultimately reducing organismal fitness. Similarly, *INSR* and *IRS1* loss-of-function mutations in hepatocytes, which result in complete insulin resistance and impaired lipogenesis, are positively selected due to strong protection against lipotoxicity. ^49,85^ However, these might not be ideal drug targets because the inhibition of insulin signaling would cause severe insulin resistance. ^106,107^ These types of conflicts underscore a central challenge of somatic genetics—the inability to predict patient-level phenotypes based on selection strength alone. *In vivo* biological exploration of a mutant clone’s cell-autonomous and non-cell-autonomous influences on organismal fitness may be an essential step. We will illustrate how modeling approaches are useful to confirm fitness changes and uncover potential discord between clone and organism.

## SYSTEMATIC TARGET DISCOVERY USING SOMATIC GENOMICS

### Somatic genomics is a new way to identify pathways with human disease relevance

Germline genetics has gained traction in the pharmaceutical industry because high-throughput approaches routinely generate impactful genotype-phenotype associations. The rapid translation of *PCSK9* from a new target identified in 1998 in familial hyperlipidemia to an approved drug helped fuel the excitement for germline genetics.^79^ Other successes include *CFTR* in cystic fibrosis^108^ and *NAV1.8* for pain.^81^ Germline genetic support for a target is associated with a 2.6-fold probability of achieving drug approval.^109^

Somatic genetics can be viewed as an alternative and complementary approach to germline genetics, whose power has been built on genomic data from more than 10 million patients. Cancer genome studies were the first large-scale somatic genomics efforts and provide a roadmap for non-malignant diseases. ^110^ Although the somatic mutation landscape in most tissues and diseases remains unexplored, initial studies suggest that somatic mutations will enrich for drug targets. ^21,23^ Using somatic genomics for target discovery has several advantages. First, it is unbiased, broad in scope, and has the potential to identify targets missed by other methods. This is because somatic mutations are less constrained by negative selection and are much more diverse than germline polymorphisms. While germline variants must be tolerated during embryogenesis and in adult tissues, somatic variants only need to be tolerated in specific tissues after development. Second, in contrast to germline genetic studies, fewer individuals may yield a similar number of findings due to the large clonal diversity within tissues. Third, mutated genes are directly selected by disease, making them relevant to the pathogenic process from which they emerge. Fourth, the approach has exquisite resolution—down to the level of individual amino acid changes. Fifth, somatic genetics is tissue- and cell-type-specific, leaving little question as to the relevant tissues or cell types involved.

### A framework to systematically identify somatic gene targets that impact disease

We propose a four-step framework to leverage somatic genomics for target discovery across a wide range of diseases (Figure 4): (1) selecting cells based on cellular and/or phenotypic markers, (2) genotyping cells using highly accurate sequencing methods, (3) deciphering patterns of selection to identify candidate genes, and (4) phenotypically validating genetic findings to nominate drug targets.

#### Step 1: Cell selection

Selecting cells of interest on the basis of cell-type, morphologic, or phenotypic markers is an important first step. Isolation of specific cell types enriches signals, which is especially important in tissues with multiple cell types. Mapping mutations to specific cell types can further support the interpretation of genetic findings, allowing the tailoring of therapeutic hypotheses to cellular processes. In tissue sections where clones are spatially defined (liver, GI tract, and skin), laser capture microdissection (LCM) followed by high-quality, low-input DNA sequencing can be used. ^14,111,112^ LCM can be guided by morphology using general hematoxylin and eosin or special stains. LCM is challenging when cell types are admixed and clonal structures cannot be resolved spatially, but mutations can be later correlated with histological features.

An alternative cell isolation method is fluorescence-activated cell or nuclei sorting (FACS or FANS). Markers are available to isolate specific cell populations with high specificity. However, clinical tissue samples are usually stored as frozen or formalin-fixed samples, which does not allow for cell dissociation. In these cases, extraction and isolation of nuclei via FANS is advantageous. Nuclear markers are available for some cell types, including neurons (NeuN) and microglia (CD68). ^113^ Recently, sorting of nuclei that were fluorescently labeled with RNA-based probes was used to isolate neuronal subtypes in the context of Huntington’s disease. ^114^ The increasing diversity of RNA-based markers obtained from single-nucleotide RNA sequencing data will allow investigators to isolate nuclei from a growing number of cell populations using FANS.

#### Step 2: Sequencing somatic mutations

Historically, technical barriers have limited discovery efforts in somatic genomics associated with normal tissues. To enable discoveries, it will be essential to conduct appropriately powered studies, taking into account the strength of selection and duration of disease exposure. While low VAF mutations can evade accurate detection by sequencing, ^7^ several factors can facilitate mutation detection. First, chronic disease provides the time required for larger clonal expansions. ^13,14,21^ Second, rapidly dividing cells shorten the time needed for clonal expansions. Third, even small clones can have large, often non-cell autonomous, contributions to the clinical phenotype. ^56^ The localization of such clones can facilitate the identification of a causative mutation. Fourth, an obvious morphological phenotype associated with mutant clones can enable the enrichment of cells with somatic mutations. While these factors can increase feasibility, evolving technical approaches described below enable sensitive and specific somatic mutation detection.

1. Bulk sequencing: traditional bulk sequencing has been sufficient to detect somatic mutations in large clones. Mutations in the blood, endometrium, and liver have been detected using approaches such as whole-exome sequencing (WES) of large tissue samples. Due to sequencing error rates, WES is generally not reliable for the detection of mutations with VAFs <5% (equating to ∼10% of diploid cells). Thus, the sequenced samples need to be clonal or oligoclonal and cannot be highly heterogeneous. Targeted sequencing with molecular barcodes allows for increased sequencing sensitivity and specificity but is limited when the genes of interest are not readily apparent.
2. Sequencing of clones derived from single cells: in tissues where cells can be isolated and cultured, clones derived from single cells (colonies or organoids) have been sequenced using standard methods, as recently demonstrated for lymphocytes. ^115^ However, these approaches lack scale and often require clonal expansion *in vitro*, which can introduce additional mutations and selection artifacts. A benefit of these methods is that high-quality whole-genome sequences can be generated from each colony, allowing the reconstruction of phylogenetic trees that illuminate the evolutionary history of somatic mutations. ^116^
3. High-accuracy sequencing: often, clonal structures are not spatially defined, and cell culture is not feasible. In these cases, high-accuracy sequencing technologies, with the requisite sensitivity to capture mutations present in single DNA molecules, have successfully been applied to a range of tissues, including post-mitotic neuronal tissue. ^113^ Modified duplex sequencing methods like NanoSeq ^8^ can achieve error rates of ∼1e− 9 errors/bp, which are ∼100 times lower than physiologic somatic mutation rates (1,000–2,500 mutations per cell, or ∼2e− 7 mutations/bp). Technologies such as NanoSeq have recently enabled high-resolution studies of the somatic mutation landscape of multiple tissues. ^117,118^
4. Single-cell sequencing: emerging technologies such as multiplexed end-tagging amplification of complementary strands (META-CS) and scNanoSeq can detect mutations in single cells, ^119–121^ but high cost and allelic dropout remain challenges. We envision that future technologies will be capable of combining single-cell DNA sequencing methods with other -omics readouts to reveal direct links between somatic mutations and their phenotypic impacts.

#### Step 3: Deciphering mutations under selection

Mutations that increase fitness lead to clonal expansions through positive selection, while those that decrease fitness will lead to negative selection. Positive selection can be detected as an excess (recurrence) of mutations in a given gene above expectation for the background mutation rate, since mutations driving clonal expansion are more frequent in the cell population than randomly occurring passenger mutations. Negative selection can be detected as depletion of functional mutations in a given gene, since cells carrying those mutations die. The major statistical challenge for these approaches is to define the expected background mutation rate. One widely used method is based on extrapolating the number of synonymous mutations within the gene or similar genes. ^122^ Other than a small fraction of synonymous mutations that affect processes such as splicing or cotranslational folding, ^122^ a conservative assumption is that the vast majority of synonymous mutations are non-functional. Accordingly, dN/dS, or the ratio of non-synonymous to synonymous mutations after correcting for their expected mutation rates, provides an accurate measurement of selection. A ratio of 1 is consistent with mutations accumulating randomly (neutrally) in the gene, whereas a ratio >1 suggests positive selection, and a ratio <1 negative selection. The approach can be applied at the level of gene sets, individual genes, domains of a gene, or even individual amino acid sites, enabling the identification of hotspot mutations. Selection signals can also be evaluated at the level of mutation types (missense or truncating) (Figure 5).

Beyond identifying genes under selection, a further challenge arises in interpretation. Positive selection on somatic mutations can occur at any stage within the life history of a clonal lineage, and the imprint of positive selection we see in the genome will represent an amalgam of the different selective forces the lineage has experienced. Thus, it becomes critical to assess whether selection on a given gene is disease-specific, as opposed to being selected with age, drug therapy, or other confounding variables. This requires mapping the somatic genome of healthy aging and other clinical contexts alongside the disease tissue. For example, to decide if a driver mutation is specific to a disease state, the frequency of the mutations as well as the dN/dS ratio in disease can be compared with the dN/dS ratio in healthy tissues. Accordingly, we calculate the difference in mutation burdens between disease and healthy tissues in terms of mutations per thousand cells (Figure 5, top panel).

In terms of therapeutic targeting, causative drivers are targets to be counteracted, protective drivers could be phenocopied, and disease-specific essential or synthetic lethal genes may offer new therapeutic vulnerabilities. Patterns of somatic mutations along the sequence of a gene can provide information about the directionality of the mutation’s effect (Figure 5, bottom). (1) Genes that have an excess of truncating mutations are likely to have lost functionality. Inhibitory molecules can mimic such loss-of-function mutations across the organ or whole body. (2) Genes that have an excess of missense mutations, potentially at the level of a single domain or site, may be activated. Agonist molecules or domain-specific modulators could mimic these potential gain-of-function effects. (3) Genes with fewer than expected missense and/or truncating mutations are under negative selection. One might consider avoiding these genes as drug targets due to potential safety concerns. It should also be emphasized that for protective mutations, putative therapies will not only target mutated cells. Instead, these mutations inspire treatments directed at all cells. Collectively, these analyses can unveil a mutation’s directional impact, the strength of selection, and cell types in which the mutations operate, altogether accelerating downstream target validation experiments.

#### Step 4: Enriching understanding of how somatic mutations impact organismal biology

While there is a successful history of developing drugs on the basis of somatic mutations in cancer, there are significant challenges associated with developing drugs based on somatic mutations in non-malignant diseases. In cancer, the goal is often to antagonize the effects of oncogenic mutations, which are compelling targets because they are implicated in cancer initiation or progression. For non-malignant disease, there is less certainty about whether or not a mutation is driving or protecting against disease. While germline genetics might reveal only the mutations that can be tolerated during development, that approach produces more information about organism-level phenotypes. For somatic mutations, increased clone fitness does not always clarify the role of the mutation in disease, and the organism-level phenotypic implications of small clones are not always apparent. Assessing a mutation’s utility as a therapeutic target requires determining if the variants increase, alter, or decrease the function of the protein, which can then help determine if mutations are causal or protective. As discussed above, mutations that can be adaptive for the cell may not be beneficial to the organ or organism. There is also the possibility that selected mutant clones are a consequence of rather than a cause of disease and thus represent epiphenomena that have little impact on disease progression. Phenotypic understanding at all biological levels (cell, tissue, organ, and organism) is needed to determine the value of the targets and whether they can be exploited to reverse disease.

Since the selection of mutant clones can be quantified using dN/dS, one might ask if it is even necessary to model mosaicism in animal models. While generating the mutation in all cells of an organ or organism could be the key biological validation step, there are reasons to model mosaicism *in vivo*. First, this can quantify the strength of selection on candidate variants, which may correlate with the organismal impact of the genetic variant. Second, even low levels of mosaicism could modulate organism-level disease phenotypes, as has been documented with CHIP, brain diseases, and fatty liver disease.^49,56,123^ Third, clonal expansion may only be occurring with specific environmental pressures (i.e., obesity, diabetes, alcohol, and smoking), and pinpointing these selective pressures can help identify the disease process in which genetic perturbation might have enhanced efficacy. For example, *Cideb* KO clones expand more dramatically in choline-deficient, highfat diets vs. western diet-fed livers.^51^ Consistently, liver-wide *CIDEB* inhibition was more protective against disease mechanisms that led to more clone expansion, suggesting that patients with more or larger *CIDEB* mutant clones might expect greater benefit from CIDEB inhibition. Understanding the relationship between clonal selection and the degree of therapeutic benefit could play a pivotal role in the future use of somatic mutations as predictive biomarkers.

A few salient animal models of mosaicism deserve mention. For CHIP, genetic variants are usually studied individually using Cre-lox, CRISPR, or bone marrow transplantation of mutant clones. ^124–126^ These types of studies have provided supportive evidence that non-cell-autonomous phenotypes (heart disease, liver disease) are in part caused by CHIP. ^35,124,126^ In the esophagus, modeling squamous cell mosaicism in mice was needed to reveal competitive interactions between *Notch1* mutant squamous cell clones and cancer cells. ^83,84,127^ The liver allows for the study of mosaic clones in a parallel, pooled fashion, where distinct variants can be compared in a side-by-side fashion. ^128^ In this way, liver mosaic models are reflective of human somatic mosaicism and permit the identification of disease/dietary variables that drive selection. There are many cutting-edge *in vivo* screening methods to generate pools of mutant cells within the liver, making this tissue an excellent testing ground for somatic genetics. ^13,85,129,130^

The above examples successfully recapitulate human somatic mosaicism in rodent models but may not always clarify the impact of perturbing individual genes in a therapeutic manner. Understanding therapeutic implications requires understanding what would happen if the mutation were present in most cells within the tissue, organ, or organism. Given potential discord between clone and organism, integrated phenotyping at multiple biological levels from cell to organism can determine if somatic mutations can be exploited to ameliorate disease. *In vitro* studies in human cells, organoids, or organs-on-a-chip can also dissect the mechanistic bases for the effects of human variants. Overexpression, base editing, or prime editing in human and animal models will also facilitate the understanding of gain-of-function variants. Another efficient approach would be to apply a tool compound in a relevant *in vivo* model of disease.

## FUTURE OUTLOOK FOR SOMATIC GENOMICS

### Somatic and germline genetic data can be integrated to nominate targets

Germline sequencing has produced large datasets, some of which are publicly available, across a multitude of traits and diseases. In the context of a strong somatic genetic signal, germline genetics could support the directionality of genetic effect, corroborate impact on a clinical phenotype, or clarify safety signals. Studying rare monogenic diseases can also help establish genotype-phenotype connections because many of these conditions have been mapped to mutations with inferred or partially characterized functional effects. Weaker germline signals could be used to bolster confidence in strong somatic signals, and vice versa. We envision the combinatorial analysis of diverse genetic modalities to assess genetic targets.

### Context-specific somatic mutations could inform therapeutic strategies

Somatic mutation patterns occurring in early vs. late stages of disease could pinpoint biology that is mechanistically relevant to that stage of disease. For example, fatty liver disease encompasses different stages, including steatosis, steatohepatitis, fibrosis, and cirrhosis. Somatic mutations that accumulate in normal livers could indicate pathways that are generically increasing clonal fitness, regardless of disease conditions. Enrichment of somatic mutations in the fibrotic stage vs. early steatotic stages could highlight cirrhosis-specific pathways. In other tissue contexts, samples taken before or after surgery could provide clues about wound healing or regeneration. In these scenarios, mutations that are enriched in regenerating vs. quiescent tissues could reveal human genetic variation in tissue repair capacity. In these ways, samples collected during routine clinical care can report broadly about diverse clinical scenarios and disease stages. Somatic genomics is poised to capitalize on these discarded but information-rich samples.

Genetic or pharmacologic pressures might also identify physiologically important pathways. Somatic mutations can be selected for or against depending on changing selective pressures. One such selective pressure comes in the form of germline mutations such as *SERPINA1* in A1AT deficiency, but somatic genetic signals could also emerge from more common genetic pressures. For example, germline *PNPLA3* polymorphisms are a common risk variant for MASLD,^131^ and somatic mutations in the diseased livers of these patients could nominate *PNPLA3*-specific rescue pathways or treatments. Another form of selective pressure might be the chronic use of medications that may lead to resistance or side effects. Some examples could be long-term use of GLP-1 agonists for obesity, immunosuppressive drugs for autoimmune disease, or immune checkpoint inhibitors for cancer. The somatic mutant clones that emerge under these selective pressures could represent resistance mechanisms that prevent efficacy or rescue mechanisms that evade side effects.

While somatic mutations are selected by clinical pressures, they might also be seen as biomarkers that report the cause of disease or predict the effectiveness of specific treatments. First, biomarkers may provide insights into the mechanism of disease, thus informing prevention or treatment strategies. Second, biomarkers can help identify the patients most likely to respond to a drug, enabling the design of smaller clinical trials with a higher probability of success. As an example, fatty livers containing more somatic mutations in *CIDEB* could predict favorable responses to CIDEB inhibition. ^51^ Third, they can provide bedside-to-bench insights that refine therapeutic hypotheses. For example, somatic mutations that emerge under therapy could provide information about resistance mechanisms, as they have in oncology and HIV treatment. ^132–134^

### Overcoming technical and biological challenges of somatic genomics

New technologies can now profile somatic genomes across larger patient populations in a cost-effective manner. Beyond sequencing, digital pathology holds promise to analyze tissues and search for linkages with somatic mutations. Mutations with strong effects could become detectable from images, highlighting regions for genotypic analysis. Once links between genotype and phenotype are established, mutant clones could be found in large digital pathology archives without genotyping. Somatic genomics requires tissue samples, but the vast array of clinically annotated tissues in large medical centers has the potential to fuel the effort. The detection of somatic mutations in peripheral blood samples holds promise for profiling larger populations, especially for immunology-focused applications. While mutant lymphoid cells are concentrated in inflamed tissues, deeper sequencing of peripheral blood could directly detect immune cell mutants. Similarly, the shedding of DNA from solid tissue into circulation has been used in oncology. Non-cancerous solid tissues shed DNA into blood, and damaged tissues with higher cellular turnover likely shed more DNA. This portends the identification of mutations from cell-free DNA (cfDNA) in lieu of tissues. This approach would be further enhanced if cfDNA could be linked to its tissue of origin, as is possible in cancer. ^135^ cfDNA could dramatically accelerate epidemiological profiling of somatic genomes, facilitating disease-specific genetics and target discovery.

Somatic genomics provides a rich opportunity for the discovery of drug targets for diseases with high unmet need. As the field matures, we are optimistic that the translational challenges discussed here will be addressed, especially because new technologies will unlock even more precise genetic insights across a larger number of diseases and patient cohorts. Despite the early stage of the field, somatic genomics has already identified compelling therapeutic targets that are selected for by disease and has the potential to revolutionize human genetics.

## Figures and Tables

**Figure 1.**
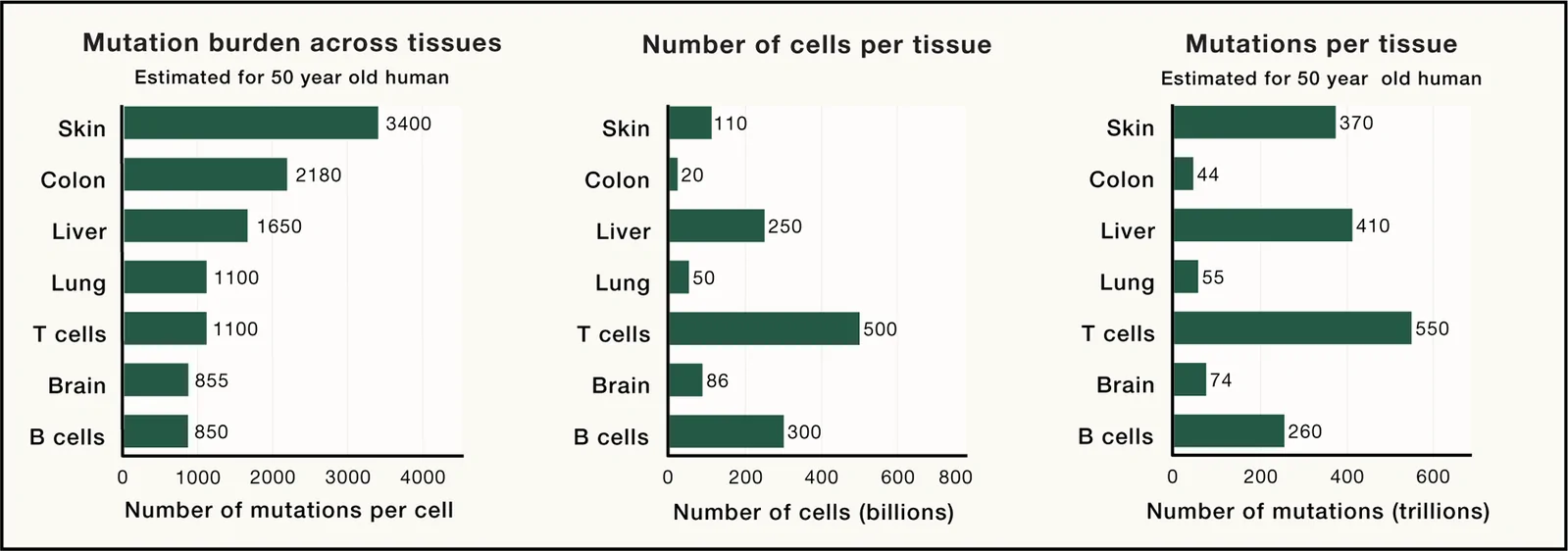
Somatic mutations are ubiquitous, permitting the sampling of most gene perturbations at the organ level The number of genes was assumed to be 20,000 (gencodegenes.org), and the fraction of coding regions in the genome was 1% (genome.gov/genetics-glossary/Gene). Mutation burden in skin was calculated based on a cohort of donors of Singaporean descent, who have lower UV-related mutation burdens. ^18^

**Figure 2.**
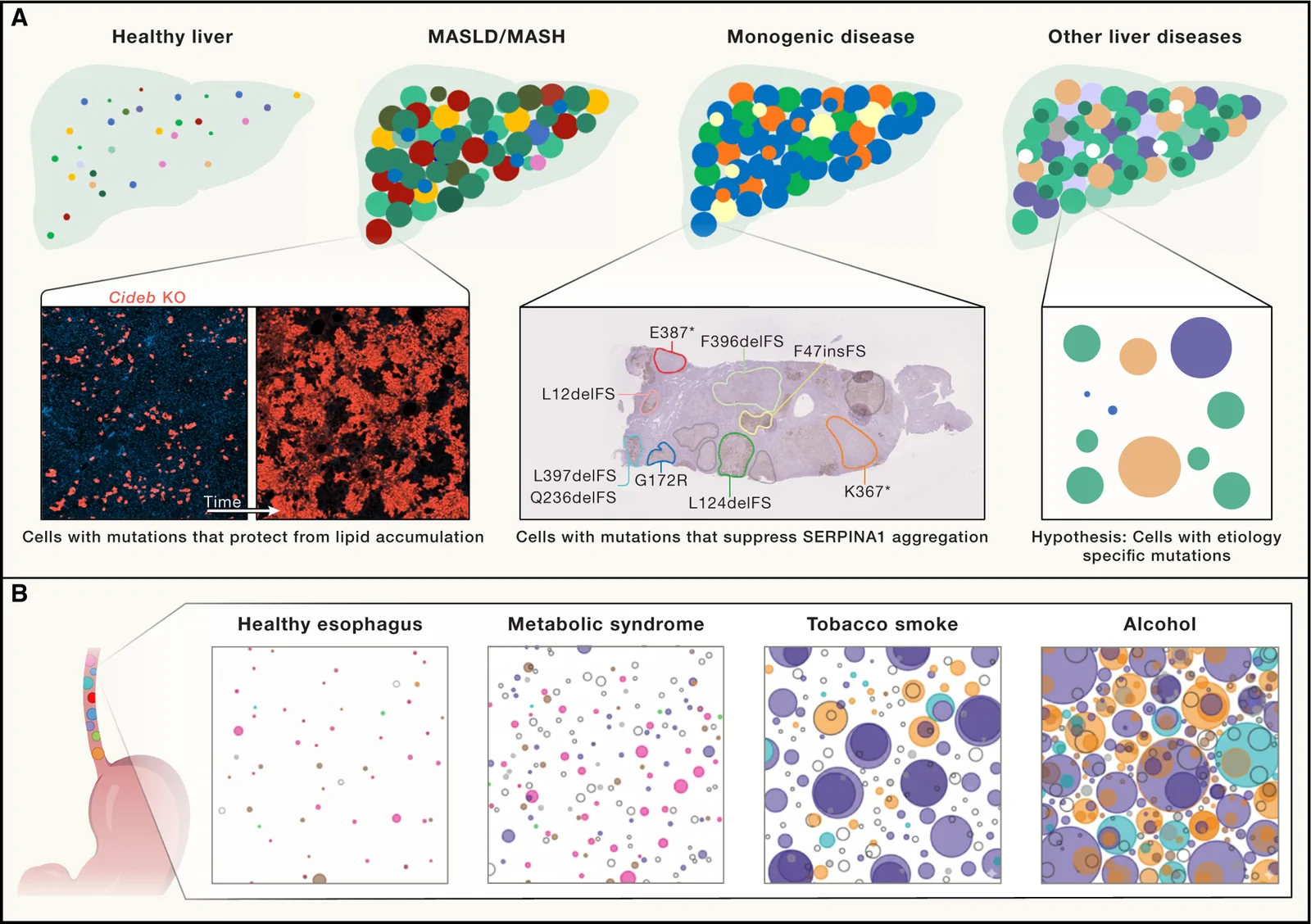
The landscape of somatic mutations is shaped by selective pressures unique to different diseases (A) The healthy liver harbors a patchwork of small clones, depicted as circles, each with its own somatic mutations. In patients with hepatic lipotoxicity from excess alcohol or carbohydrate consumption, mutations that impair *de novo* lipogenesis will protect hepatocytes. These will preferentially survive and expand to repopulate the damaged liver. In a patient with alpha-1-antitrypsin deficiency, somatic mutations in the *SERPINA1* gene can alleviate liver damage by preventing misfolded SERPINA1 protein from aggregating, thus improving liver cell survival. (B) In the esophagus, even healthy individuals harbor somatic mutations, most prominently in *NOTCH1*/*2*, *PIK3CA*, and *TP53*. Clones with these mutations can expand in the context of metabolic syndrome, tobacco use, or excessive alcohol.

**Figure 3.**
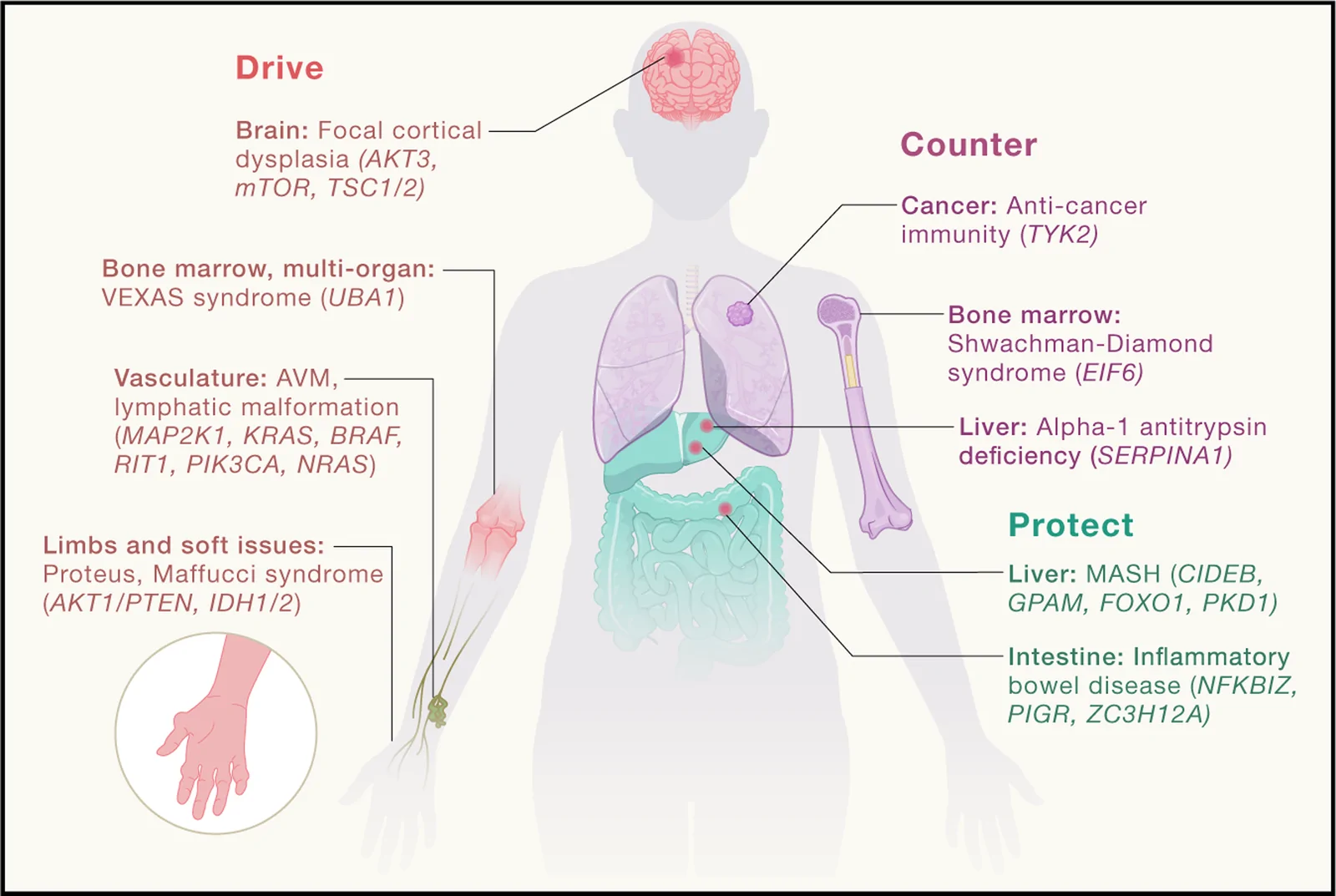
Somatic genomics uncovers the outcomes of evolutionary competitions within our tissues, which can drive disease, counter monogenic disease, or protect from common diseases

**Figure 4.**
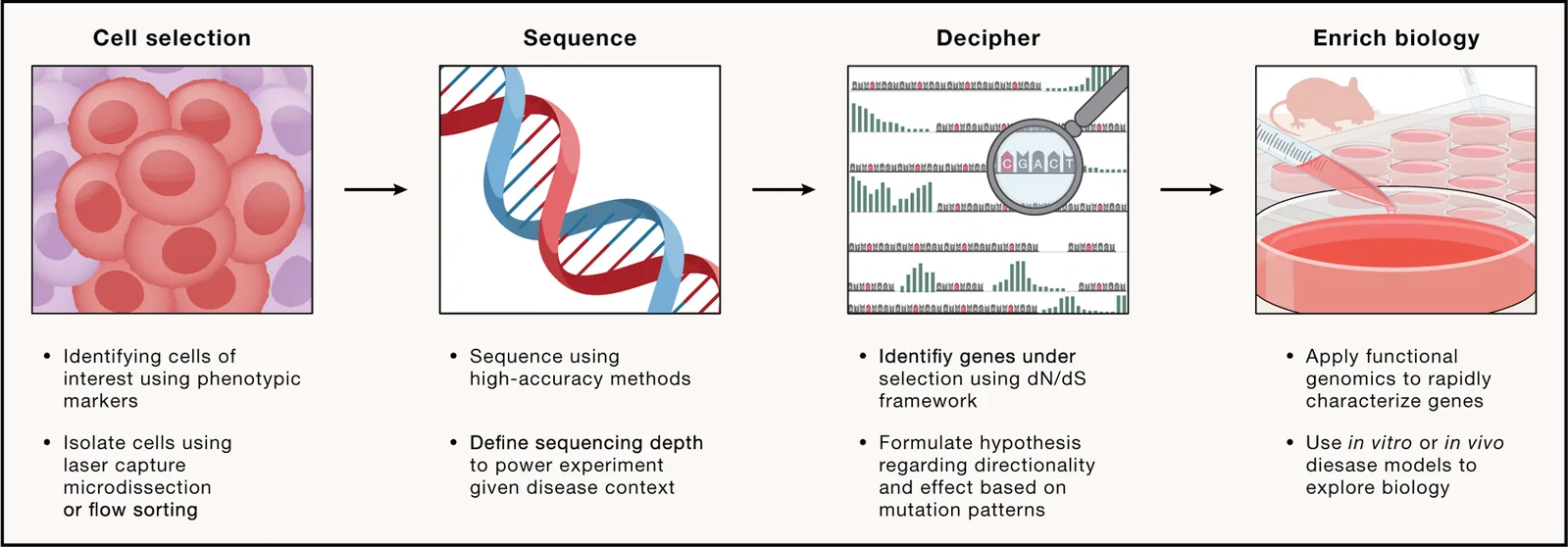
A four-step framework to systematically identify somatic gene targets that impact disease and inform therapeutic strategies

**Figure 5.**
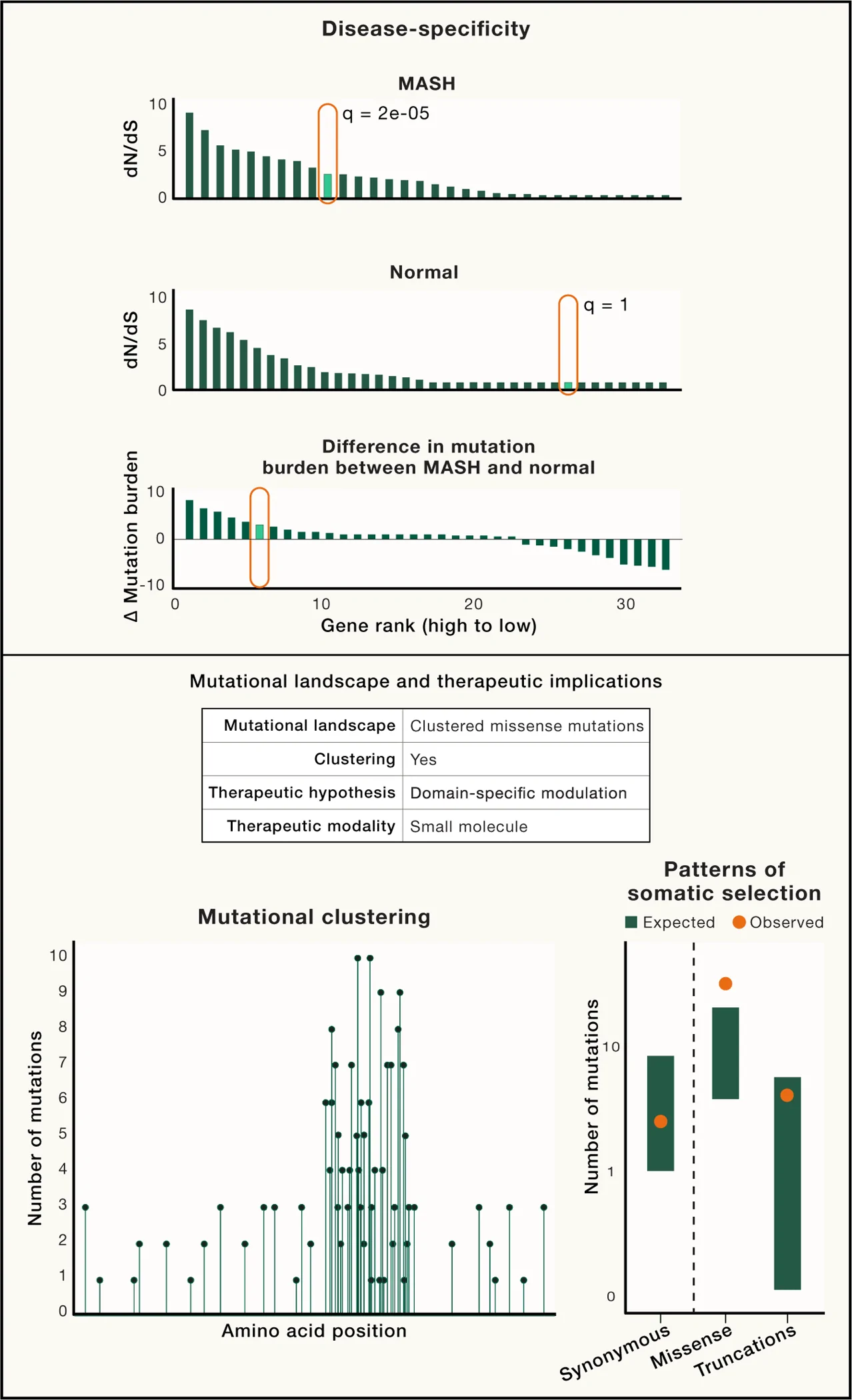
Patterns of somatic selection and clustering of mutations can inform therapeutic hypotheses An increased frequency of mutations in the disease vs. normal state would suggest that the mutant phenotype is specifically selected for in the context of that disease and thus more therapeutically relevant in the disease scenario (top). Mutation distribution provides additional clues about gene and mutation function. Truncating mutations that occur without patterns of clustering would indicate an inhibitory effect, whereas clustered missense mutations would suggest that a specific protein domain has functional relevance (bottom left). A statistical excess of missense mutations can indicate mutations that activate gene function, whereas an excess of truncating mutations indicates inhibition (bottom right). Gene-level *p* values were computed using dNdScv, and *q* values were obtained by Benjamini-Hochberg false discovery rate (FDR) adjustment across all genes tested.

**Table 1.** Illustrative list of genes under selection in different organ and disease contexts

Tissue	Disease	Gene symbols	Translational hypotheses and insights

Brain	Focal cortical dysplasia	*AKT3, MTORC1, TSC1, TSC2*	Translational hypothesis: mTOR inhibition with everolimus will reduce seizures caused by somatic mutations in the brain (NCT03198949)
Bone marrow	Vacuoles, E1 enzyme, X-linked, autoinflammatory, somatic syndrome (VEXAS)	*UBA1*	Translational hypothesis: rescue of UBA1 activity will lead to disease-modifying benefits in VEXAS
Vascular and lymphatic	Arteriovenous and lymphatic malformations	*MAP2K1, KRAS, BRAF, RIT1, PIK3CA, NRAS*	Translational hypothesis: RAS, MAPK, MEK, or PI3K inhibition will reduce AVMs (NCT05983159)
Limb	Soft tissue overgrowths (Proteus and Maffucci syndromes)	*AKT1, PTEN, IDH1/2*	AKT1 inhibition was tested in Proteus syndrome patients (NCT03094832); positive clinical responses to IDH1 inhibitors have been reported in enchondromas
Multiple cancer types	Anti-cancer immunity	*TYK2*	Translational hypothesis: activation of TYK2 will increase anti-tumor immunogenicity
Bone marrow	Shwachman-Diamond syndrome (SDS)	*EIF6*	Translational hypothesis: reduction in EIF6 dose or activity will l ead to disease-modifying benefits in SDS
Liver	Alpha-1 antitrypsin deficiency (AATD)	*SERPINA1*	Translational hypothesis: therapeutic targeting of C terminus of alpha-1-antitrypsin will lead to chain truncation and disease-modifying benefits in A1AD
Liver	Metabolic-dysfunction-associated steatohepatitis (MASH)	*CIDEB, GPAM, FOXO1, PKD1*	Translational hypothesis: inhibition of *CIDEB* or *GPAM* or targeting *FOXO1* at site S22 will benefit MASH patients; CIDEB-siRNA is being tested clinically (NCT06836609)
Intestine	Inflammatory bowel disease (IBD)	*NFKBIZ, PIGR, ZC3H12A*	The translational hypothesis that IL-17 inhibitors will alleviate colitis was disproved clinically, highlighting the need for additional somatic genetic evidence

Interpretation of mutation patterns from somatic genomics enables the formulation of therapeutic hypotheses.
